# Sonic Hedgehog Ligand: A Role in Formation of a Mesenchymal Niche in Human Pancreatic Ductal Adenocarcinoma

**DOI:** 10.3390/cells8050424

**Published:** 2019-05-08

**Authors:** Francesca Saini, Richard H. Argent, Anna M. Grabowska

**Affiliations:** Ex Vivo Cancer Pharmacology Centre of Excellence, Cancer Biology, Division of Cancer and Stem Cells, School of Medicine, University of Nottingham, Nottingham NG7 2RD, UK; sainifrancesca@hotmail.com (F.S.); richard.argent@nottingham.ac.uk (R.H.A.)

**Keywords:** pancreatic ductal adenocarcinoma (PDAC), sonic hedgehog (Shh), tumour microenvironment

## Abstract

Pancreatic ductal adenocarcinoma (PDAC) is characterised by desmoplasia, thought to support progression and chemotherapeutic resistance. The Hedgehog pathway is known to play an important role in this cancer. While the upregulation of Sonic hedgehog (Shh) in the epithelium of PDAC is known, we investigated its expression in the tumour microenvironment in order to find new targets for new chemotherapeutical approaches. Immunohistochemistry was used for the investigation of Shh and Vimentin in primary human pancreatic tissues. Gene (qRT-PCR) and protein (immunofluorescence) expression of Shh, αSMA (a marker of the mesenchymal phenotype) and periostin (a marker of mesenchymal cells within a mixed population) were investigated in in vitro cell models. Shh expression was significantly upregulated in the stromal and epithelial compartments of poorly-differentiated PDAC samples, with a strong correlation with the amount of stroma present. Characterisation of stromal cells showed that there was expression of Shh ligand in a mixed population comprising αSMA^+^ myofibroblasts and αSMA^−^ mesenchymal stem cells. Moreover, we demonstrated the interaction between these cell lines by showing a higher rate of mesenchymal cell proliferation and the upregulation of periostin. Therefore, targeting stromal Shh could affect the equilibrium of the tumour microenvironment and its contribution to tumour growth.

## 1. Introduction

Pancreatic cancer is one of the most aggressive solid tumours, with only a 6% 5-year survival rate. Diagnosis is frequently late and, in advanced tumours, chemotherapy is a palliative measure that increases survival by only a few weeks or months [1,2]. Even when identified at a relatively early stage, intervention to prevent progression and metastasis (mainly surgery and chemotherapy) is usually ineffective. Pancreatic cancer is characterised by a strong desmoplastic reaction, which is known to support pancreatic adenocarcinoma (PDAC) progression and metastasis [3,4,5] and contributes to chemotherapeutic resistance, either acting as a physical barrier to drug delivery or supporting tumour cell growth [6,7]. Stromal cells in PDAC include a variety of cell types such as myofibroblasts (also called cancer-associated fibroblasts, CAFs), fibrocytes, pericytes and pancreatic stellate cells (PSCs) as well as endothelial and immune cells [4,8]. An important role for myofibroblasts in the modulation of stromal physiology and pathology in the cancer setting, through the secretion of chemokines, cytokines, matrix metalloproteinases (MMPs) and extracellular matrix (ECM) components has been demonstrated [9]. Myofibroblasts, in general, express α-smooth muscle actin (αSMA), as well as vimentin, desmin, cadherin11 and collagen type 1; however, due to their diverse origins, including trans-differentiation from adipocytes, epithelial and endothelial cells, or differentiation from mesenchymal stem cells (MSCs) [9,10] that account for approximately 20% of myofibroblasts in the tumour microenvironment [9,11,12], it has proven difficult to define a set of markers that can be used to robustly define them [10,13].

MSCs and myofibroblast cells are also the two populations that constitute the bone marrow-derived mesenchymal stem cell niche (mSCN) that originates from the differentiation of the stem cells (MSCs) into niche cells (myofibroblasts). The concept of the mSCN involves interaction between these two populations responsible for maintaining, by direct cell contact or paracrine signalling, proliferation, survival, and the self-renewing capacity of stem cells [14]. There is a key role for the mSCN, recruited from the bone marrow into the tumour microenvironment, in cancer growth and progression to metastasis [12,15,16,17]; for example, when chronic gastritis, metaplasia and dysplasia were induced by *Helicobacter felis* infection, or by constitutive expression of IL-1β in the stomach of αSMA-RFP+ transgenic mice (in which expression of RFP was regulated by the αSMA promoter), increased numbers of αSMA positive cells (myofibroblast cells) were observed in both mouse models at the later stage of dysplasia [12]. The myofibroblast cells originating in the infected mice in culture were often surrounded by RFP-negative cells (undifferentiated MSCs) suggesting the existence of an in vitro mixed population. Bone marrow-derived myofibroblasts/CAFs were longer-lived and promoted tumour growth significantly more than other stromal cells [12]. Consistent with the idea of the role of MSC and αSMA^+^ myofibroblasts in tumour progression, tail vein injection of bone marrow-derived MSCs in mice with partial pancreatectomy resulted in recruitment of these cells to the pancreas and their differentiation into PSCs (cells expressing αSMA with similar features of myofibroblast cells), pancreatic ductal epithelial cells, and vascular endothelial cells strongly contributing to the regeneration of the pancreas [18]. 

Reactivation and overexpression of the Hedgehog (Hh) pathway [19,20,21,22,23,24,25,26,27,28,29] plays a key role in the development, progression and the promotion of the desmoplastic reaction in pancreatic cancer [5,30,31] as well as other tumours. In some of these tumours, paracrine activation of stromal cells by the Hh ligand (Shh) released by epithelial cancer cells has been observed [30,31,32]. Interestingly, the Hh pathway is also involved in the interplay between stem and niche cells. However, contrasting theories describe its role in the niche via an effect on cell differentiation into myofibroblast cells [5,33,34] or, in contrast, via self-renewal and proliferation of stem cells [35,36,37]. The idea of Hh ligand-induced myofibroblast differentiation is supported by in vitro assays in which rat hepatic stellate cells (HSCs; the liver analogue of PSCs) in CCL4-cirrhosis in vivo models and in vitro cell culture with serum showed Shh and Gli2 (one of the Hh pathway transcription factors) expression, loss of Hh pathway inhibition, and upregulation of myofibroblast marker expression (αSMA expression, Collagen1β, and mesenchymal-associated transcription factors Lhx2 and Msx2). Consistently, acquisition of the mesenchymal phenotype was inhibited by treatment with cyclopamine, an antagonist of the Hh pathway [38]. 

Thus, upregulation of *SHH* gene expression during MSC differentiation into myofibroblast-like cells (αSMA protein and gene expression upregulation) can occur in the absence of epithelial cells [38] when a mixed population, consisting of both bone marrow-derived MSCs and myofibroblast cells, rather than either cell-type alone, is present [12].

In this study, we hypothesise that Shh, the ligand of the Hedgehog pathway, is upregulated only in a mixed mesenchymal population comprising αSMA^+^ and αSMA^−^ cells, as previously shown in chronic gastritis, metaplasia, and dysplasia [12]. To test this hypothesis, this study compared primary human PDAC to normal pancreatic tissues and used in vitro cells models of αSMA positive and negative mixed populations. We demonstrated that the Shh ligand is not just expressed by epithelial cells, as previously demonstrated, but at the stromal level too in the advanced stages of PDAC. Moreover, we showed that Shh and periostin are upregulated in an αSMA^+^/αSMA^−^ mixed population suggesting an interaction between the two populations resulting in the formation of a stem cell niche in the tumour microenvironment of PDAC which potentially drives the desmoplastic reaction associated with this disease. Targeting this niche with anti-Shh therapy could alone, or in combination with anti-cancer cell drugs, provide a novel approach to PDAC treatment.

## 2. Materials and Methods

### 2.1. Primary Pancreatic Tissues

Twenty pancreatic tissue samples were obtained from patients undergoing resection for pancreatic tumours in Queen’s Medical Centre, Nottingham, UK with informed patient consent and with full ethical approval (MREC reference H0403/37). Matched normal tissues were taken from the same patients away from the tumour without affecting resection margins. Moreover, based on their grade of differentiation, an expert histopathologist classified the tumour tissues as moderately (MDT) or poorly (PDT) differentiated PDAC and confirmed the normal samples as non-cancerous. Samples were snap frozen in liquid nitrogen or fixed in 4% formaldehyde as soon as they were received.. 

### 2.2. Immunohistochemistry and Immunocytochemistry

For immunohistochemistry (IHC), fixed tissues were embedded in paraffin and cut into 4 µm sections, dewaxed, and blocked for endogenous peroxidase activity using 1% hydrogen peroxide in methanol for 15 min (Shh staining) or 3% hydrogen peroxide in distilled water (vimentin staining). Antigen retrieval was performed in 10 mM citric acid pH 6.0 at 98 °C for 20 min. Sections were incubated for 30 min in 20% blocking serum and 1% bovine serum albumin (BSA) in PBS. Sections were incubated with primary antibodies diluted in PBS (Shh 1:100 (Abcam, Cambridge, UK); vimentin 1:50 (Dako, Ely, UK)). Negative controls were run in parallel. Sections were rinsed in PBS and then incubated with a biotinylated secondary antibody for 30 min, before incubation with avidin-biotin complex (Vector Laboratories, Peterborough, UK) for 20 min and visualisation using 3,3’-diaminobenzidine (Dako). 

Staining was quantified using H-score analysis of six randomly-chosen images, calculated as: (3× percentage of high intensity) + (2× percentage of medium intensity) + (1× percentage of low intensity), giving a range from 0–300. A semi-automated system written in Qwin standard (Leica microsystem, Wetzlar and Mannheim, Germany) was used to quantify sections. Six randomly-chosen images of each tissue were also examined for percentage Shh protein expression in the stromal and epithelial compartments assessed by eye.

For immunofluorescence analysis, cells were fixed in 4% paraformaldehyde for 30 min at room temperature, washed in PBS and 4 × 10^4^ cells cytospun onto polylysine-coated slides using a Thermo Scientific Shandon Cytospin and blocked in PBS containing 0.1% Tween-20, 1% BSA, 10% normal serum and 0.3 M glycine, for 1 h, then incubated for 16 h at 4 °C with 100 µL primary antibody diluted in PBS containing 1% BSA. The following primary antibodies were used: Rat anti-human Shh monoclonal (1:25, Abcam); mouse anti-human αSMA clone 1A4 (1:100, Sigma-Aldrich, Poole, UK); rabbit anti-human periostin polyclonal (1:100, Abcam).

Negative controls were carried out in parallel by substituting the primary antibody with an isotype control. Cells were washed and incubated with AlexaFluor488-conjugated secondary antibodies diluted in PBS containing 1% BSA, and mounted with ProLong^®^ Gold Antifade reagent with DAPI (ThermoFisher Scientific). Immunofluorescence staining was quantified by analysing three randomly-chosen images per slide. Each image was divided into 4 equal-sized regions of interest (ROI) and a final H-score (one for each ROI, 4 per image) calculated. The H-score values were then ranked as 0–100 = 1, 101–200 = 2; 201–300 = 3.

### 2.3. In Vitro Cell Cultures and Stem Cell Niche Models

Pancreatic CAFs were isolated from primary tumour tissues by digesting minced tissue with 0.4 U/mL dispase and 120 U/mL collagenase (ThermoFisher Scientific, Paisley, UK) at 37 °C and culturing in PCA medium (DMEM supplemented with 7.2 μg/mL insulin (Sigma-Aldrich), 1 μg/mL hydrocortisone (Sigma-Aldrich), 100 U/mL penicillin, 100 U/mL streptomycin, 0.25 μg/mL amphotericin B, 2 mM L-glutamine, and 20% FBS). Human MSCs were purchased from ScienCell (Carlsbad, CA, USA) and cultured in mesenchymal stem cell media (MSCM, ScienCell) which consists of basal medium 5% FBS and Mesenchymal Stem Cell Growth Supplement (ScienCell). 

### 2.4. Stem Cell Niche In Vitro Models

A mixed population of αSMA positive and negative cells was obtained by culturing MSCs in ‘free differentiation medium’ (FDM; DMEM containing 10% FBS) for the length of the experiment. A single population of MSCs was maintained by culturing MSCs at low passage (P2) in ‘preventing differentiation medium’ (PDM; MSCM containing 5% FBS) for the length of the experiment. Mixed mesenchymal populations of MSCs with CAFs or myofibroblast (MF)-like cells were obtained by co-culturing 1.8 × 10^5^ total cells in a 1:1 ratio. MF-like cells were obtained by culturing MSCs in MSCM lacking the growth factor supplements, or by treating them with 10 ng/mL TGFβ (Humanzyme, Chicago, IL, USA) for 1 week.

### 2.5. Three-Dimensional Tumour Growth Assay (3D-TGA)

MSCs and/or CAFs from a grade 3 PDAC tumour sample were embedded in 3 mg/mL Cultrex basement membrane extract (Trevigen, Gaithersburg, MD, USA) diluted in RPMI-1640 medium [39] alone or at a 1:1 ratio. Briefly, 5 × 10^3^ mesenchymal cells were mixed with an equal volume 6 mg/mL ice-cold Cultrex and 25 µL plated into pre-warmed 384-well plates in quintuplicate and incubated at 37 °C in standard cell culture conditions. The AlamarBlue assay (Thermo Fisher Scientific) was used (10%, 37 °C 1 h) to monitor cell growth immediately after plating (day 0) and daily from day 3 onwards, using a FlexStation II (Molecular Devices, Sunnyvale, CA, USA) fluorescent plate reader.

### 2.6. RNA Extraction, cDNA Synthesis, and Gene Expression Analysis

Total RNA was extracted from cells using TRI reagent (Sigma-Aldrich), and from tissue samples using the AllPrep DNA/RNA Micro kit (Qiagen, Crawley, UK). RNA samples were reverse transcribed into cDNA using Superscript II Reverse Transcriptase (ThermoFisher Scientific). Gene expression analysis was performed by q-RT-PCR using a StepOnePlus machine (ThermoFisher Scientific) using SYBR Green, 5 µM each primer, and the following conditions: Denaturation at 95 °C for 10 min, followed by 40 cycles of 95 °C for 15 s and 60 °C for 60 s, and a final melt curve stage at 95 °C for 15 s, 60 °C for 1 min and 95 °C for 15 min. Primers sequences were as follows: *HPRT:* Sense (5′-ATTATGCTGAGGATTTGGAAAGGG), antisense (5′-GCCTCCCATCTCCTTCATCAC); *SHH*: Sense (5′-CGACCGCAGCAAGTACGGCA), antisense (5′-CGATTTGGCCGCCACCGAGT); *ACTA2*: Sense (5′-AACATGGCATCATCACCAACTG), antisense (5′-GCAACACGAAGCTCATTGTAGAA); *POSTN*: Sense (5′-ACACACCCGTGAGGAAGTTGC), antisense (5′-TGAGAACGACCTTCCCTTAATCGTC).

### 2.7. Statistical Analysis

Data were tested for normal distribution using the D’Agostino & Pearson omnibus test. The analysis of the differences between groups of data was performed using one- or two-way ANOVA for normally-distributed data, or the Kruskal and Wallis test for non-parametric data. In the case of comparison between only two groups, a t-test for paired or unpaired data was considered. Correlation analysis was performed using Spearman and Pearson tests for non-parametric and parametric data distribution, respectively.

## 3. Results

### 3.1. Shh Is Overexpressed in Both the Stromal and Epithelial Compartments of Advanced Pancreatic Tumours

To investigate the expression of the Hedgehog pathway ligand, Shh, in Pancreatic Ductal Adenocarcinoma (PDAC) and its relationship with tumour progression, the expression of Shh was examined in normal and tumour pancreatic tissue including both poorly and moderately-differentiated PDAC.

**Figure 1:**
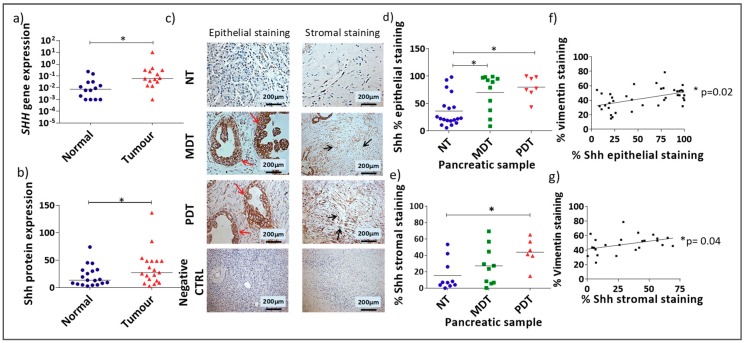
Shh is overexpressed in PDAC in the epithelial and stromal compartments: (**a**) *SHH* gene expression in tumour tissues compared to paired normal tissues (* *p* < 0.05 Wilcoxon matched-pairs signed rank test). (**b**) Quantification of IHC staining (H-score) for Shh protein expression in normal and tumour tissues (* *p* < 0.05 Wilcoxon matched-pairs signed rank test). (**c**) Example images of Shh IHC staining in normal pancreatic tissue (NT), and moderately differentiated pancreatic tumour (MDT), and poorly differentiated pancreatic tumour (PDT) tissues. Negative controls demonstrate the absence of staining either in normal tissue (column on the left) or PDT tissue (column on the right) when an isotype control antibody was substituted for the specific antibody to the target. Red arrows show epithelial Shh expression and black arrows show stromal Shh expression. Semi-quantitative analysis of the percentage Shh staining in the epithelial (**d**) and stromal (**e**) compartments (* *p* = < 0.05 Kruskal-Wallis multiple comparison Test) for NT, MDT, or PDT samples. Analysis of correlation (Spearman correlation test) between % vimentin staining and % epithelial (* *p* = 0.015) (**f**) or stromal (* *p* = 0.04) (**g**) Shh staining.

**Figure A1:**
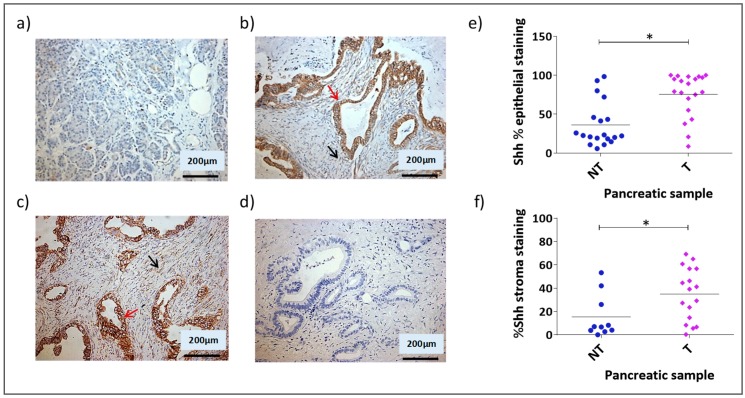
Shh expression in normal and tumour pancreatic tissues. 19 paired normal and tumour primary pancreatic tissues and 1 unpaired tumour primary pancreatic tissue (39 total samples) were investigated for expression of the Hh pathway ligand, Shh by IHC analysis. Based on histophatological analysis the tumour tissues were divided into moderate (MDT) and poorly differentiated tumour (PDT). Representative images of Shh staining are shown: (**a**) normal pancreatic tissue; (**b**) moderately differentiated pancreatic tumour; **(c**) poorly differentiated pancreatic tumour; (**d**) negative control demonstrates absence of staining either in MDT tissue when an isotype control antibody was substituted for the specific antibody to the target. Red arrows and black arrows indicate epithelial and stromal cancer cells expressing Shh, respectively. Semi-quantitative analysis of the percentage Shh staining in the epithelial (**e**) and stromal (**f**) compartments in all tumour samples (* ***p*** < 0.05 Kruskal-Wallis multiple comparison Test).

**Figure A2:**
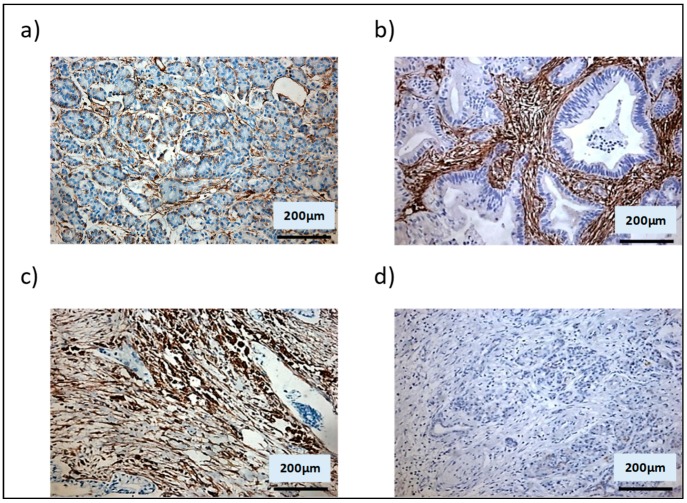
Vimentin expression in human pancreatic primary tissues. Example images of IHC analysis of vimentin protein expression in: (**a**) normal pancreatic human tissues, (**b**) moderately differentiated pancreatic tumour and (**c**) poorly differentiated pancreatic human tumour, (**d**) Negative control demonstrating absence of staining in PDT tissue when an isotype control antibody was substituted for the specific antibody to the target.

Shh gene (qRT-PCR) and protein expression analysis (IHC staining) showed significantly higher expression of Shh in pancreatic tumour compared with matched normal tissue (Figure 1a,b, Wilcoxon test for paired samples). Examination of Shh expression in the epithelial and stromal compartments showed that there was significantly higher Shh expression in both compartments in PDACs in comparison to the normal samples (Figure 1c, Supporting Figure A1). Moreover, while Shh expression was significantly higher in the epithelial compartment of both moderately differentiated tissues (MDT) and poorly differentiated tissues (PDT) compared with normal tissue (Figure 1d), in the stromal compartment Shh was only significantly higher in PDTs (Figure 1e). Furthermore, there was a strong correlation between vimentin (Supporting Figure A2), used as a marker of stromal content, and Shh expression in both the epithelial and stromal compartments (Figure 1f,g) supporting the idea of a possible contribution of Shh signalling to the stromal expansion in pancreatic cancer [5] and indicating Shh as a new possible target to reduce the stromal mass in this cancer.

### 3.2. Shh Is Upregulated in a Mixed Population of αSMA^−^ and αSMA^+^ Cells

**Figure 2:**
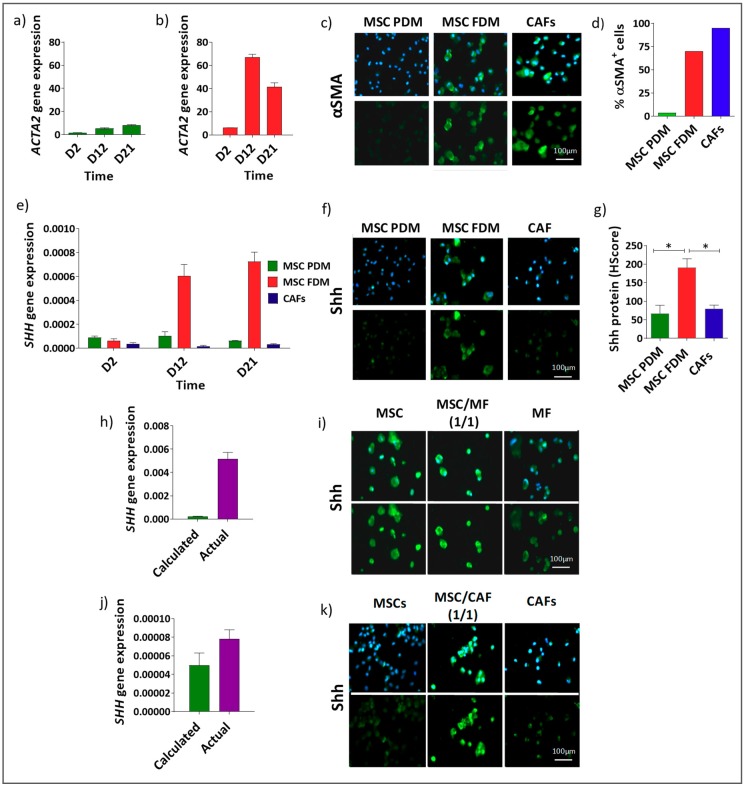
(**a**–**d**) Shh is upregulated in mixed populations of αSMA^+^ and αSMA^−^ mesenchymal cells: All the representative images in this figure (**c,f,i,k**) show αSMA^−^ cells in the column on the left; αSMA^+^/αSMA^−^ mixed population in the central column and αSMA^+^ cells in the column on the right. *ACTA2* gene expression in MSCs grown in PDM (**a**) and FDM (**b**). (**c**) Representative images of αSMA expression in MSCs grown in PDM (αSMA^−^ cells) or FDM (αSMA^+^/αSMA^−^ mixed population), or CAFs grown in FDM (αSMA^+^ cells). (**d**) Percentage αSMA^+^ and αSMA^−^ cells after 21 days of culture. Analysis of Shh gene (**e**) and protein (**f**,**g**) expression in the same 3 cell models (* *p* < 0.05 one-way ANOVA multiple comparison test). (**h**–**k**) Shh expression in in vitro models of αSMA^−^/αSMA^+^ mixed populations. αSMA^−^ cells (MSCs) were mixed at a 1:1 ratio respectively with two different αSMA^+^ population (MSCs treated with TGFβ) (**h**,**i**) and CAFs (**j**,**k**). Data were analysed by comparing the actual *SHH* gene expression detected in these in vitro mixed population models with the calculated average of *SHH* gene expressions observed in the αSMA^−^ and αSMA^+^ cells grown separately in the same experiment. Gene (**h**,**j**) and protein (**i**,**k**) expression confirmed the increased expression of Shh in αSMA^−^/αSMA^+^ mixed populations.

The observation that Shh was expressed in the stroma of PDAC samples led us to assess the ability of specific stromal cells, in particular mesenchymal cells, to express Shh. Human bone marrow-derived MSCs and CAFs were investigated for Shh expression at the gene and protein levels (Figure 2). MSCs were cultured in a “preventing differentiation medium” (PDM) to maintain them in their inactivated and undifferentiated state (as determined by αSMA expression [9]) or cultured in a “free differentiation medium” (FDM) to allow them to differentiate. MSCs transferred from PDM to FDM, but not when maintained in PDM, displayed increased levels of *ACTA2* expression (Figure 2a,b) changing their percentage of αSMA^+^ cells from 3% to 70% and resulting in a mixed population of αSMA^+^ and αSMA^−^ cells at a 70:30 ratio. CAFs, in FDM, had a high percentage of αSMA^+^ cells (95%) compared with MSCs maintained in PDM in which only 3% of cells were αSMA^+^ (Figure 2c,d). Similar percentages were obtained in previous work on gastric cancer [12], supporting our results.

**Figure A3:**
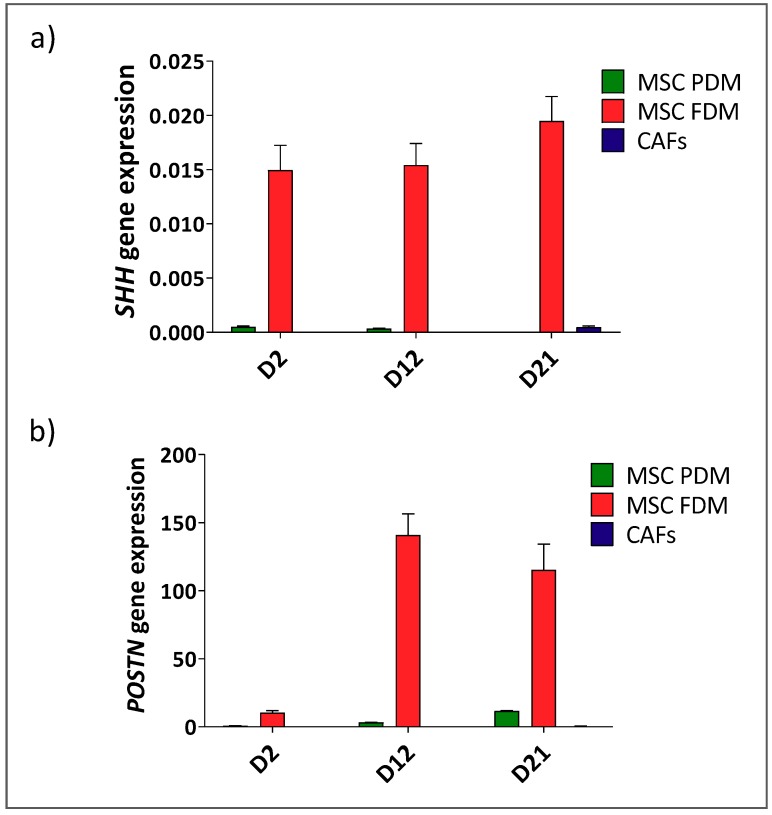
*SHH* and *POSTN* gene expression in in vitro models consisting of αSMA^−^ and αSMA^+^ mixed populations in comparison with models consisting of either αSMA^−^ or αSMA^−^ cells alone. *SHH* (**a**) and *POSTN* (**b**) gene expression in MSCs grown in PDM (green) or FDM (red), or in CAFs (blue).

Thus, three models were available: CAFs cultured in FDM, comprising mostly αSMA^+^ cells; MSCs cultured in PDM, where most cells were αSMA^−^; and MSCs cultured in FDM, forming a mixed population of αSMA^+^ and αSMA^−^ cells (70:30 ratio). Shh gene and protein expression were assessed in these three models (Figure 2e–g). Shh was elevated in the mixed population, coinciding with the upregulation in αSMA expression observed after culturing for 12 days in FDM (Figure 2a,e) but not in CAFs or MSCs cultured solely in PDM. Similar patterns were observed in independent replicate experiments (Supporting Figure A3).

To further investigate Shh as a marker of a αSMA^+^/αSMA^−^ mixed population, the effect of a mixture of αSMA^+^ and αSMA^−^ cells was analysed in two further models using MSCs maintained in PDM mixed with two different αSMA^+^ populations: MSCs treated with TGFβ (Figure 2h,i), or CAFs (Figure 2j,k). In order to show the effect of the co-culture, the gene expression measured (actual gene expression) in the mixed populations was compared with the expression that would be anticipated if the co-culture had no effect on gene expression (calculated expression, i.e., by calculating the average expression of the two individual populations of cells if mixed together and no change in gene expression occurred as a result). *Shh* gene (Figure 2h,j) and protein expression (Figure 2i,k) levels were again increased in the mixed populations in comparison to Shh expression in the individual cell-types.

### 3.3. αSMA^+^ and αSMA^−^ Cells Interact Between Each Other in the Shh Expressing Mixed Population

To further characterise the mixed population models described above and investigate the interaction between the two population that constitute the mixed models, they were assessed for expression of the marker periostin (Postn), an adhesion molecule observed to be overexpressed only in the context of a mixed population comprising cancer stem cells and αSMA^+^ cells as response of the interplay between these two populations [16,40,41].

**Figure 3:**
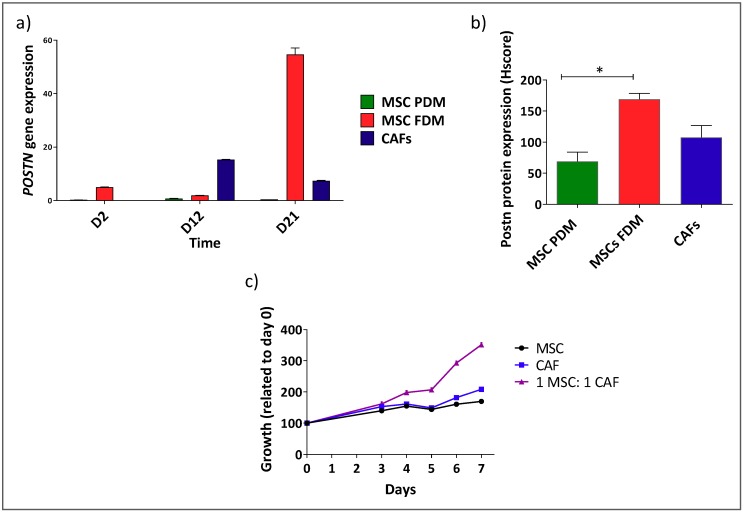
In vitro models comprising mixed populations of αSMA^+^ and αSMA^−^ cells show interaction between the two component cell lines. Postn expression in MSCs grown in FDM at the gene (**a**) and protein level (* *p* < 0.05 one-way ANOVA multiple comparison test) (**b**) in comparison to MSCs grown in PDM or CAFs. (**c**) Growth of αSMA^−^ (MSCs in PDM) and αSMA^+^ (CAFs) mixed at a 1:1 ratio or each population alone in a 3D assay.

Postn gene and protein expression levels were increased in MSCs cultured in FDM for 21 days (mixed αSMA^+^/αSMA^−^ cells) in comparison to MSCs cultured in PDM (αSMA^−^) and CAFs cultured in FDM (αSMA^+^) (Figure 3a,b, Supporting Figure A3), indicating that there is an interaction between the two populations that constitute these mixed population models. In addition, a marked increase in growth was observed when MSCs (αSMA^−^) and CAFs (αSMA^+^) were mixed in a 1:1 ratio and cultured in a 3D in vitro model, compared with the growth of either population alone, again, confirming an interaction between the two cell types that influences their phenotype (Figure 3c).

## 4. Discussion

In this study, we have demonstrated the upregulation of the Hh pathway ligand, Shh, in the stromal compartment in pancreatic cancer, in particular during the advanced stage. Importantly, we show that this upregulation of stromal Shh expression is dependent on the presence of a mixed population of both αSMA^+^ and αSMA^−^ cells in which periostin is also upregulated, and mesenchymal cell growth is accelerated, thus further supporting the concept of the formation of a mesenchymal niche, with a phenotype different from that of either of the αSMA^+^ or αSMA^−^ cells alone, that could contribute to pancreatic cancer progression. Moreover, we also highlight potential roles for stromal Shh as a marker of poorly differentiated pancreatic cancer and as a potential chemotherapeutic target to reduce and block the integrity of the pancreatic cancer stroma and its ability to sustain growth and metastasis.

Upregulation of Shh in pancreatic cancer compared with normal tissue has been previously described [30,41], and the Shh pathway is known to have a key role in pancreatic tumour development, progression, and metastasis [23,42,43,44]. However, our observation of Shh expression at the stromal level has only been previously observed in haematological malignancies [20,45], and in a small number of studies in mouse model solid tumours [12,36,46,47]. Recent studies suggest a paracrine model for Hh signalling in certain cancers in contrast with the previous finding of tumour epithelial cells responding to Hh ligand overexpression in an autocrine manner [19,41,43,48,49]. Previous studies have also shown the expression of Shh by IHC in primary human tissues, demonstrating paracrine signalling of the Hh pathway in human samples [41,43,50], however, none of them demonstrated expression of the Hh ligand in the stromal cells or further investigated its potential role in this context as indicated in our study.

We only observed formation of a stable mixed population of αSMA^−^ and αSMA^+^ cells (30%/70% ratio) and upregulation of Shh when human bone marrow-derived MSCs were cultured in DMEM supplemented with 10% FBS and not when MSCM was used, the latter containing growth factors that prevent MSC activation and upregulation of αSMA. The requirement for mixed αSMA^−^/αSMA^+^ cells was demonstrated by showing that Shh expression was low in either αSMA^−^ or αSMA^+^ cells (CAFs and MSCs, respectively) cultured alone. Previously, expression of Shh has been observed when quiescent HSCs or PSCs, multipotent cells involved in liver and pancreatic fibrosis, were cultured in DMEM containing 10% FBS [38,51]. In these conditions quiescent HSCs were shown to acquire a mesenchymal phenotype and become activated, expressing αSMA, collagen and mesenchyme-associated transcription factors Lhx2 and Msx2, a process which was Hh pathway-dependent [38]. Upregulation of Shh at the gene level has also previously been observed in an in vitro model based on mouse MSCs comprising a mixed population of αSMA^−^/αSMA^+^ similar to those in our study [12], supporting our gene and protein observations in human MSC-based models. In the future, it will be important to determine whether Shh expression is upregulated in both the αSMA^−^ and αSMA^+^ or only in one of these, and to further dissect out the mechanism underlying the paracrine signalling between the two populations of cells that leads to this outcome.

Periostin expression was investigated based on its role as an adhesion molecule expressed in αSMA^+^ myofibroblast-like cells and present in a niche containing cancer stem cells with a metastatic phenotype [16], suggesting a role for mesenchymal cells expressing this marker in driving the late stages of cancer progression. In line with this, periostin expression was higher in the mixed population in comparison to the αSMA^−^ and the αSMA^+^ single populations in our study.

Using a 3D in vitro model that mimics the in vivo tumour microenvironment, we showed the higher proliferative power of αSMA^−^/αSMA^+^ mixed population in comparison to the αSMA^+^ CAF and αSMA^−^ MSC single populations, further suggesting an interplay between these two populations. A number of studies have confirmed the strong desmoplastic effect in pancreatic tumour beginning during the pancreatitis stage and becoming more marked by the time full adenocarcinoma has developed [4,52,53,54]. This change is believed to be a consequence of the interplay between epithelial cancer cells and PSCs that are quiescent in normal tissues. The progression of cancer triggers signalling pathways, including the Hh pathway, which activate PSCs (inducing their proliferation) and elicits the proliferation of other resident fibroblasts and the recruitment of MSCs from bone marrow, leading to an overall increase of the tumour stromal component characterised by a tumour–specific gene expression signature [4,5,52,53,55,56]. A duplex effect of the Hh pathway either in pancreatic cancer or in pancreatitis has been demonstrated [57,58]. Genetic or treatment-induced Hh pathway activation in engineered pancreatic cancer mouse models induced the desmoplastic reaction typical of this tumour but also reduced pancreatic tumour cell proliferation. Moreover, inhibition of Hh pathway in the same models showed reduction of the stromal mass but increased the growth of tumour cells [58]. Consistently, the Hh pathway showed a protective effect in acute pancreatitis where the stromal mass is low and instead showed an important role in sustaining, and inducing, the progression of chronic pancreatitis where there is a consistent fibrotic mass [57]. Furthermore, a direct role of Hh in promoting fibrosis by recruiting pancreatic stellate cells has been demonstrated [56]. In line with this, mouse models with chronic pancreatitis and PSC in vitro treated with the natural anthraquinone Rhein showed a decreased expression of TGFβ, fibronectin-1, collagen-α1 and Shh [51].

These studies highlight the important role of the Hh pathway in maintaining the integrity of the tumour microenvironment and in regulating the balance between epithelial tumour cells and stromal mass in pancreatic cancer.

Our results taken together suggest a new interpretation of the role of the Hh pathway in pancreatic cancer, not just as a pathway reactivated in the cancer epithelium but as an autocrine signal in the tumour microenvironment, as recently observed and demonstrated in gastric cancer [46]. Moreover, we suggest that the expression of Shh observed in this study in the advanced stages of human pancreatic primary tissue could potentially be a marker of the presence of an αSMA^−^/αSMA^+^ mixed population which expresses molecules associated with the cancer stem cell niche and with driving metastatic potential [16] in the pancreatic tumour microenvironment.

The role of the tumour microenvironment in the resistance to chemotherapy, as well as the role of the Hh pathway, are topics of complex and often contrasting conclusions [59]. The effect of the tumour microenvironment on chemoresistance to gemcitabine (a drug used to treat PDAC) [59] has been argued from results showing a protective effect of αSMA^+^ stromal cells on hepatocellular carcinoma and pancreatic cancer through their association with vascularisation [60]. Moreover, clinical trials on the use of Hh inhibitors are still not exhaustive, and in some cases, showed failure [61]. On the other hand, the combination of gemcitabine and Hh pathway inhibitors, e.g., the IPI-29 Hh inhibitor, which reduces the stromal component in PDAC mouse models, has given encouraging results [62,63,64].

Our results open the possibility that the Shh ligand is a new important target in the stromal context and suggests a potential mechanism underlying the encouraging results obtained when Hh inhibitors are combined with standard-of-care. Targeting Shh could, in fact, affect the equilibrium of the stroma tumour microenvironment that makes an important contribution to tumour growth and survival [12,46].

## 5. Conclusions

We have demonstrated that Shh and periostin are upregulated as a result of interaction between αSMA^−^ and αSMA^+^ stromal cells. This formation of a Shh-expressing mesenchymal niche may be involved in driving the desmoplastic response and drug resistance characteristic of PDAC, and could explain the encouraging findings observed when Hh inhibitors have been combined with chemotherapeutic agents.

## Abbreviations

MSCs	mesenchymal stem cells
αSMA	alpha-smooth muscle actin-expressing
mSCN	mesenchymal stem cell niche
Shh	sonic hedgehog
PDAC	pancreatic adenocarcinoma
CAFs	cancer-associated fibroblasts
PSCs	pancreatic stellate cells
MMPs	matrix metalloproteinases
ECM	extracellular matrix
MDT	moderately differentiated tumour
PDT	poorly differentiated tumour
ROI	regions of interest
FDM	free differentiation medium
PDM	preventing differentiation medium
3D-TGA	three-dimensional tumour growth assay
qRT-PCR	quantitative real-time polymerase chain reaction
IHC	immunohistochemistry
